# A vertigo network derived from human brain lesions and brain stimulation

**DOI:** 10.1093/braincomms/fcad071

**Published:** 2023-03-17

**Authors:** Yanran Li, Lei Qi, Frédéric L W V J Schaper, Di Wu, Maximilian Friedrich, Jialin Du, Tao Yu, Qiao Wang, Xiaopeng Wang, Di Wang, Guangyuan Jin, Aihua Liu, Chunqiu Fan, Yuping Wang, Michael D Fox, Liankun Ren

**Affiliations:** Department of Neurology, Clinical Center for Epilepsy, Xuanwu Hospital, Capital Medical University, National Center for Neurological Disorders, Beijing 100053, China; Department of Neurology, Clinical Center for Epilepsy, Xuanwu Hospital, Capital Medical University, National Center for Neurological Disorders, Beijing 100053, China; Center for Brain Circuit Therapeutics, Department of Neurology, Brigham and Women’s Hospital, Harvard Medical School, Boston, MA 02115, USA; Department of Psychiatry, Brigham and Women’s Hospital, Harvard Medical School, Boston, MA 02115, USA; Department of Radiology, Brigham and Women’s Hospital, Harvard Medical School, Boston, MA 02115, USA; Department of Neurology, Clinical Center for Epilepsy, Xuanwu Hospital, Capital Medical University, National Center for Neurological Disorders, Beijing 100053, China; Center for Brain Circuit Therapeutics, Department of Neurology, Brigham and Women’s Hospital, Harvard Medical School, Boston, MA 02115, USA; Department of Psychiatry, Brigham and Women’s Hospital, Harvard Medical School, Boston, MA 02115, USA; Department of Radiology, Brigham and Women’s Hospital, Harvard Medical School, Boston, MA 02115, USA; Department of Neurology, University Hospital Wuerzburg, Josef-Schneider Strasse 11, Wuerzburg 97080, Germany; Department of Neurology, Clinical Center for Epilepsy, Xuanwu Hospital, Capital Medical University, National Center for Neurological Disorders, Beijing 100053, China; Department of Pharmacy Phase I Clinical Trial Center, Xuanwu Hospital, Capital Medical University, Beijing 100053, China; Department of Functional Neurosurgery, Beijing Institute of Functional Neurosurgery, Xuanwu Hospital, Clinical Center for Epilepsy, Capital Medical University, Beijing 100053, China; Department of Neurology, Clinical Center for Epilepsy, Xuanwu Hospital, Capital Medical University, National Center for Neurological Disorders, Beijing 100053, China; Department of Neurology, Clinical Center for Epilepsy, Xuanwu Hospital, Capital Medical University, National Center for Neurological Disorders, Beijing 100053, China; School of Engineering Medicine, Beihang University, Beijing 100191, China; Department of Neurology, Clinical Center for Epilepsy, Xuanwu Hospital, Capital Medical University, National Center for Neurological Disorders, Beijing 100053, China; Department of Neurology, Clinical Center for Epilepsy, Xuanwu Hospital, Capital Medical University, National Center for Neurological Disorders, Beijing 100053, China; Department of Neurology, Clinical Center for Epilepsy, Xuanwu Hospital, Capital Medical University, National Center for Neurological Disorders, Beijing 100053, China; Department of Neurology, Clinical Center for Epilepsy, Xuanwu Hospital, Capital Medical University, National Center for Neurological Disorders, Beijing 100053, China; Center for Brain Circuit Therapeutics, Department of Neurology, Brigham and Women’s Hospital, Harvard Medical School, Boston, MA 02115, USA; Department of Psychiatry, Brigham and Women’s Hospital, Harvard Medical School, Boston, MA 02115, USA; Department of Radiology, Brigham and Women’s Hospital, Harvard Medical School, Boston, MA 02115, USA; Athinoula A. Martino’s Center for Biomedical Imaging, Departments of Neurology and Radiology, Massachusetts General Hospital, Boston, MA 02114, USA; Department of Neurology, Clinical Center for Epilepsy, Xuanwu Hospital, Capital Medical University, National Center for Neurological Disorders, Beijing 100053, China; Chinese Institute for Brain Research, Beijing 102206, China

**Keywords:** vertigo, lesion network mapping, direct cortical stimulation

## Abstract

Vertigo is a common neurological complaint, which can result in significant morbidity and decreased quality of life. While pathology to peripheral and subtentorial brain structures is a well-established cause of vertigo, cortical lesions have also been linked to vertigo and may lend insight into relevant neuroanatomy. Here, we investigate the supratentorial lesion locations associated with vertigo and test whether they map to a common brain network. We performed a systematic literature search and identified 23 cases of supratentorial brain lesions associated with vertigo. We mapped the lesion locations to a standard brain template and computed the network of brain regions functionally connected to each lesion location, using a ‘wiring diagram’ of the human brain termed the human connectome (*n* = 1000). Sensitivity was assessed by identifying the most common connection to lesion locations associated with vertigo, and specificity was assessed through comparison with control lesions associated with symptoms other than vertigo (*n* = 68). We found that functional connectivity between lesion locations and the bilateral ventral posterior insula was both sensitive (22/23 lesions) and specific (voxel-wise family-wise error-corrected *P* < 0.05) for lesion-induced vertigo. We computed connectivity with this hub region to define a lesion-based vertigo network, which included regions in the bilateral insula, somatosensory cortex, higher-level visual areas, cingulate sulcus, thalamus and multiple cerebellar regions in the territory of the posterior inferior cerebellar artery. Next, we used stereo-electroencephalography (80 stimulation sites across 17 patients) to test whether stimulation sites associated with vertigo mapped to this same network. We found that 36/42 (86%) of stimulation sites eliciting vertigo fell within the lesion-based vertigo network in contrast to 16/39 (41%) of stimulation sites that did not elicit vertigo. Connectivity between stimulation sites and our lesion-based hub in the ventral posterior insula was also significantly associated with vertigo (*P* < 0.0001). We conclude that cortical lesions and direct electrical stimulation sites associated with vertigo map to a common brain network, offering insights into the causal neuroanatomical substrate of vertigo.

## Teaser

Vertigo is a common neurological complaint, affecting almost all people at some point during their life span. Li *et al*. showed a cortical vertigo network derived from brain lesions and the intracranial direct electrical stimulation sites, which offers new insights into the neuroanatomical substrate of vertigo.

## Graphical Abstract

**Graphical Abstract:**
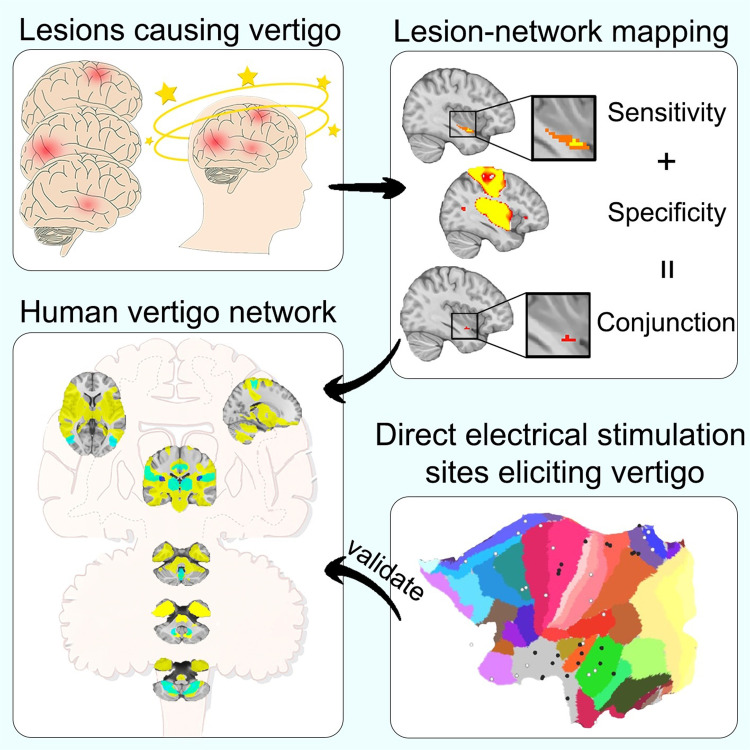

## Introduction

Vertigo is a common neurological complaint resulting in significant morbidity, repeated medical attendance and decreased quality of life.^1^ Vertigo is defined as a subjective sensation of imbalance including the illusive perception of the rotating or tilting of self despite no motion occurring.^2^ Conditions causing vertigo are typically linked to pathology involving the vestibular labyrinth and vestibular brain circuits in the cerebellum and brainstem. Vertigo arising from lesions involving the cerebral cortex is uncommon—and when present primarily due to epileptic activity or due to electrocortical activation—but there are case reports of spontaneous reports of vertigo in association with cortical lesions.^3,4^ Although these cases are relatively rare, they may lend important insight into the neuroanatomy of vertigo including involvement of specific cortical regions or networks.

Studies in non-human primates and functional neuroimaging in humans have identified cortical regions thought to play a role in vestibular processing, referred to as the ‘vestibular cortex’. In non-human primates, vestibular cortex is located in the midposterior Sylvian fissure and consists of the parieto-insular vestibular cortex (PIVC) and visual posterior Sylvian (VPS).^5,6^ The human homologues of these regions are thought to be located in the parietal opercula 2 (OP2)^7^ and posterior insular cortex (PIC).^6,8^ Lesions and direct electrical stimulation (DES) to the human vestibular cortex have been associated with vertigo, but vertigo can also occur following lesions and stimulation to cortex outside of these regions.^3,4,9^ Why some cortical lesion locations or stimulation sites result in vertigo while others do not remain unclear, but it is an active topic of investigation.^10^

Mounting evidence suggests that brain lesions causing a specific neurological symptom are more likely to be mapped to a brain network than to a single brain region.^11^ A recently developed technique, termed lesion network mapping, can test whether different brain lesion locations causing a specific symptom map to a common brain network.^12^ This method has successfully been used to identify brain networks associated with lesion-induced parkinsonism, dystonia, amnaesia, depression and mania among others.^13,14^ Here, we use this technique to test whether heterogeneous supratentorial lesion locations associated with vertigo map to a common brain network. We then test our lesion-based results against brain stimulation sites associated with vertigo, an approach referred to as convergent causal mapping.^15^

## Materials and methods

### Case selection

Case reports describing vertigo following a focal brain lesion were systematically reviewed from published literature. We searched PubMed for articles describing human subjects written in English with the terms: (vertigo) AND {[(stroke) OR haemorrhage] OR ischemic} according to the Preferred Reporting Items for Systematic Reviews and Meta-Analyses guidelines.^16^ Inclusion criteria were (i) a clear description of rotatory vertigo with the feeling of spinning of either the body or the surroundings; (ii) vertigo resulting from an acute stroke; (iii) lesions causing isolated vertigo on supratentorial area; and (iv) a published figure showing the location of the lesion. Exclusion criteria were (i) vertigo caused by multiple lesions or not limited lesions; (ii) lesions could not be reliably localized because of poor image quality; (iii) subtentorial lesions; and (iv) non-rotatory vertigo.

### Lesion locations

Lesion locations, displayed in original publications, were manually traced onto a standardized brain template [0.5 × 0.5 × 0.5 mm, Montreal Neurological Institute (MNI) 152 2009b] on the basis of neuroanatomical landmarks using ITK-SNAP (www.itksnap.org) software. Of note, this approach that provided an approximation of 3D lesion has been consistently validated its sufficiency for lesion network mapping by prior works.^17,18^

In order to relate these lesion locations to the classic vestibular cortex, we generated the region of interest (ROI) for OP2 and PIC. An OP2 region, the homologue area of PIVC in monkeys, was extracted from the Julich Atlas because this atlas is the only available parcellation that includes a cytoarchitectonic parcellation of OP2.^19^ PIC was extracted from Brainnetome Atlas in which it is parcellated as rostroventral area of the supramarginal gyrus.^6,20^

### Lesion network mapping

To determine the network of brain regions functionally connected to each lesion location, we conducted the connectivity analysis in MNI152 space with 2 × 2 × 2 mm voxel size resolution using a resting state functional MRI data set collected from 1000 healthy adult subjects.^21^ A Fisher’s *Z* transformation was applied to normalize the distribution of values for each of the 1000 functional connectivity correlation maps. A *T*-map for each lesion network was computed with a *T*-score value for each individual voxel. This *T*-score represents the statistical significance of each voxel connectivity to the lesion location. Each lesion network was subsequently thresholded at *T* = 7 to create a binarized map [corresponding to voxel-wise family-wise error (FWE)-corrected *P* < 10^−6^] of brain regions connected to the lesion location. This threshold is consistent with prior publications.^13,22^ To ensure that our results were not dependent on the *T*-threshold, we repeated our analysis with *T*-thresholds of five (voxel-wise FWE-corrected *P* < 0.05), nine (voxel-wise FWE-corrected *P* < 10^−9^) and eleven (voxel-wise FWE-corrected *P* < 10^−11^). Finally, all the binarized maps of each lesion were overlapped to identify regions with shared connectivity.

### Specificity testing

To test for specificity, unthresholded lesion network maps of all vertigo lesions were compared with the unthresholded lesion network maps from control lesions causing other neurological syndromes (*n* = 68) by means of two-sample *t*-test (voxel-wise FWE-corrected *P* < 0.05). For control lesions not associated with vertigo, 20 lesions causing facial palsy, 17 lesions causing amnaesia,^14^ 19 lesions causing mania and 12 lesions causing Parkinson’s disease from previous works were included.^13^

### Defining a lesion-based vertigo network

The conjunction analysis was performed to identify the network positive hub, which was retained by multiplying the sensitivity (lesion network mapping) analysis and the specificity testing. By definition, connectivity with positive hub identified by conjunction analysis of sensitivity and specificity test defined a distributed brain network encompassing the lesion locations causing vertigo while avoiding control lesions.

### DES sites eliciting vertigo

We reviewed all the recordings of the DES mapping performed in 165 patients who underwent stereo-electroencephalography (SEEG) in the epilepsy centre of Xuanwu Hospital between 1 January 2016 and 20 December 2019. In all patients, invasive investigation was determined because of insufficient information to define the epileptogenic zone after non-invasive investigation during presurgical evaluation by multidisciplinary team. The trajectories and target sites for implantation of SEEG electrodes were designed on the basis of clinical ground. SEEG electrodes included semi-rigid steel electrodes with 8–16 contacts (contact one is the most distal contact), 2 mm in length, 0.8 mm in diameter and 1.5 mm apart. The procedure of DES was performed after capturing habitual clinical seizures during chronic video monitoring, which has been described in our previous studies.^23,24^ Briefly, patients were instructed to sit in semi-supine position in bed and report any symptoms or feelings during the DES processing. Stimulation at 50 Hz (pulse duration 0.2 ms, biphasic rectangular stimuli of alternating polarity) lasting for 3 s was applied to two serials adjacent contacts over the cortex, which was considered exciting the cortex in prior work.^25^ Each stimulation settings and clinical findings were recorded on a standardized form, and all stimulation procedures were videotaped. When patients reported they felt spinning of either the body or the surroundings, they were considered to experience vertigo and the corresponding stimulation sites were selected.

This study was approved by the Institutional Review Board Committee in accordance with the ethical standards of the Declaration of Helsinki, and informed consent was obtained from all patients and/or guardians.

### SEEG electrode localization

To calculate the coordinates of stimulation sites causing vertigo in the standardized MNI152 stereotactic space, SEEG electrodes were reconstructed. Firstly, postoperative 3D CT images were linearly co-registered to the preoperative MRI individually using Statistical Parametric Mapping (https://www.fil.ion.ucl.ac.uk/spm/). The results of co-registrations were manually adjusted and refined for each patient if needed. Then, using Advanced Normalization Tools (http://stnava.github.io/ANTs/), the MRI was non-linearly normalized into the MNI space to reconstruct the lead trajectory (Supplementary Fig. 4). Both the linear co-registration and non-linear normalization were processed using Lead-DBS toolbox (www.lead-dbs.org). For localizing each stimulation site, the coordinates of the individual contact on each electrode were computed.

### Comparison of the lesion-based vertigo network with DES network mapping

For visualization, the DES sites eliciting and not eliciting vertigo were displaying on Brodmann areas using Connectome Workbench software (https://www.humanconnectome.org/software/connectome-workbench)^26^. The relation of the generated lesion-based vertigo network to stimulation sites eliciting vertigo was firstly evaluated. Then, identical to the lesions, functional connectivity with each stimulation site was defined across 1000 healthy subjects and analysed as described above. Sensitivity map was obtained as the most common regions functionally connecting each stimulation sites eliciting vertigo. Specificity map was calculated by comparing stimulation sites eliciting vertigo with control sites that did not elicit vertigo from the same patients using two-sample *t*-test. The relation of lesion-based vertigo network was further compared with the sensitivity and specificity map derived from stimulation sites, respectively.

### Statistical analysis

The statistical analysis in the process of lesion network mapping and specificity testing was described in detail in the “Materials and methods” section. To compare the functional connectivity with the positive hub of the human vertigo network between regions eliciting vertigo and not eliciting vertigo, one-tailed two-sample *t*-test was used. The alpha level (*P* < 0.05) was used to determine significance.

## Results

### Lesion locations associated with vertigo

**Figure 1:**
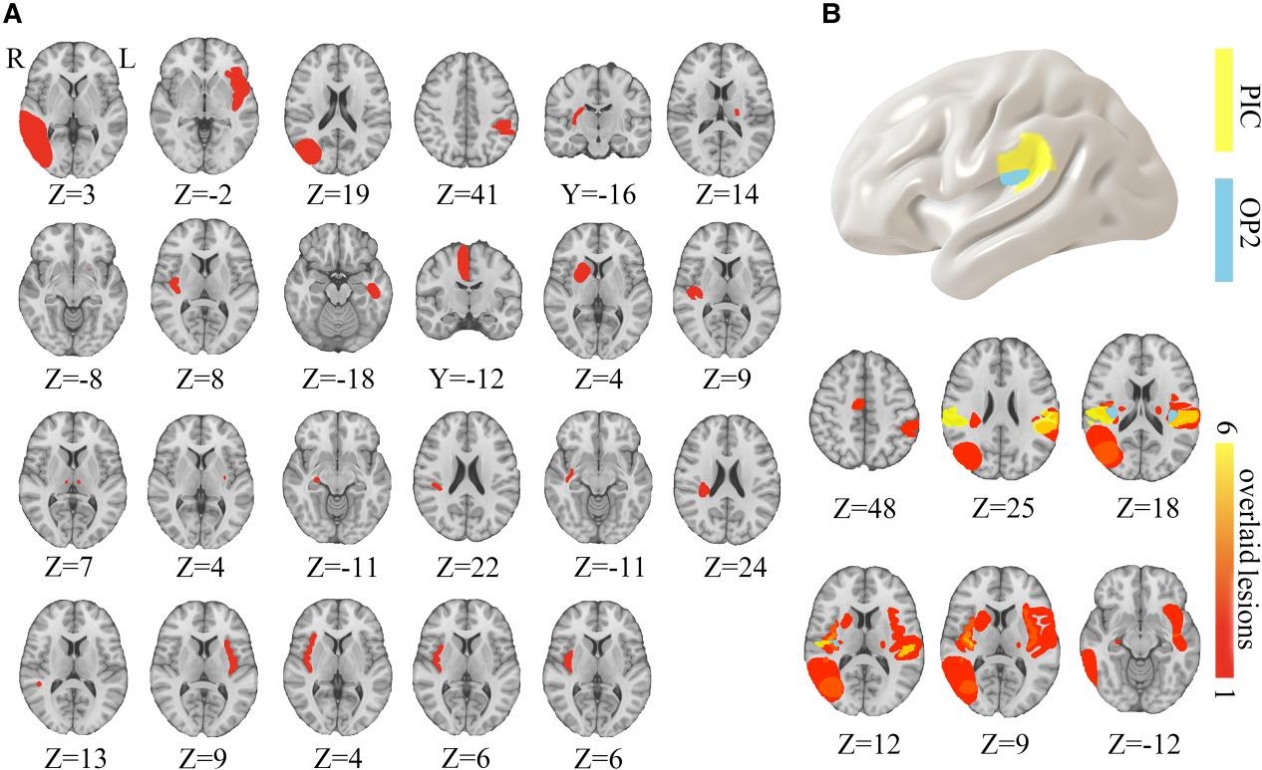
**Lesion locations associated with vertigo.** (**A**) Lesion locations, numbered 1 through 23, were manually traced onto a standardized brain template (0.5 × 0.5 × 0.5 mm, MNI152 2009). (**B**) Overlap of lesion locations causing vertigo in relation to traditional vestibular cortex in the PIC and OP2. The upper panel showed the locations of PIC (yellow) and OP2 (azure), while the lower panel illustrate the lesions’ locations overlap (red-yellow) causing vertigo. PIC, posterior insular cortex; OP2 = parietal opercula 2.

Twenty-three patients (10 males and 13 females) with a documented temporal relationship between acute supratentorial stroke and vertigo were identified (Supplementary Table 1, Supplementary Fig. 1). The original descriptions of vertigo from these patients are shown in Supplementary Table 2. Lesion locations were spatially heterogeneous (Fig. 1A) and spanned multiple different brain regions including the insular cortex (*n* = 9), the temporal lobe/hippocampus (*n* = 4), the parietal lobe (*n* = 4), the basal ganglia (*n* = 2), bilateral thalami (*n* = 1), internal capsule (*n* = 2) and supplementary motor area (*n* = 1). Fourteen of these lesions were separated from the traditional vestibular cortex of the PIC and OP2 (Fig. 1B).

### Lesion network mapping of vertigo

While lesion locations associated with vertigo were heterogeneously distributed across the brain, 96% (22/23) of these lesion locations were positively connected to the bilateral ventral posterior insula with *T*-threshold at seven (corresponding to voxel-wise FWE-corrected *P* < 10^−6^). The peak lesion network overlap in the posterior insula was constant and independent of the *T*-threshold at five (voxel-wise FWE-corrected *P* < 0.05), nine (voxel-wise FWE-corrected *P* < 10^−9^) and eleven (voxel-wise FWE-corrected *P* < 10^−11^) (Supplementary Fig. 2).

**Figure 2:**
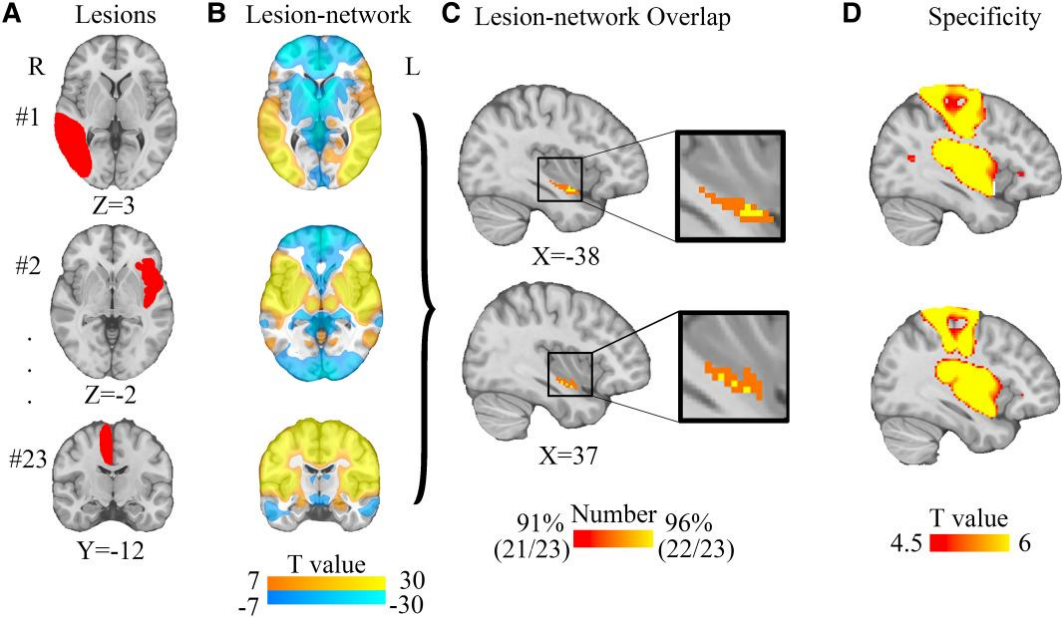
**Lesion network mapping of vertigo.** (**A**) Representative lesion locations causing vertigo. (**B**) Lesion networks, representing regions functionally connected to lesion locations causing vertigo, were computed using a normative connectome (*n* = 1000). (**C**) Lesion network overlap (sensitivity testing) showing regions connected to most (22/23, 96%) lesion locations. (**D**) Specificity testing, comparing lesion networks of lesions associated with vertigo versus lesions not associated with vertigo (voxel-wise FWE-corrected *P* < 0.05).

**Figure 3:**
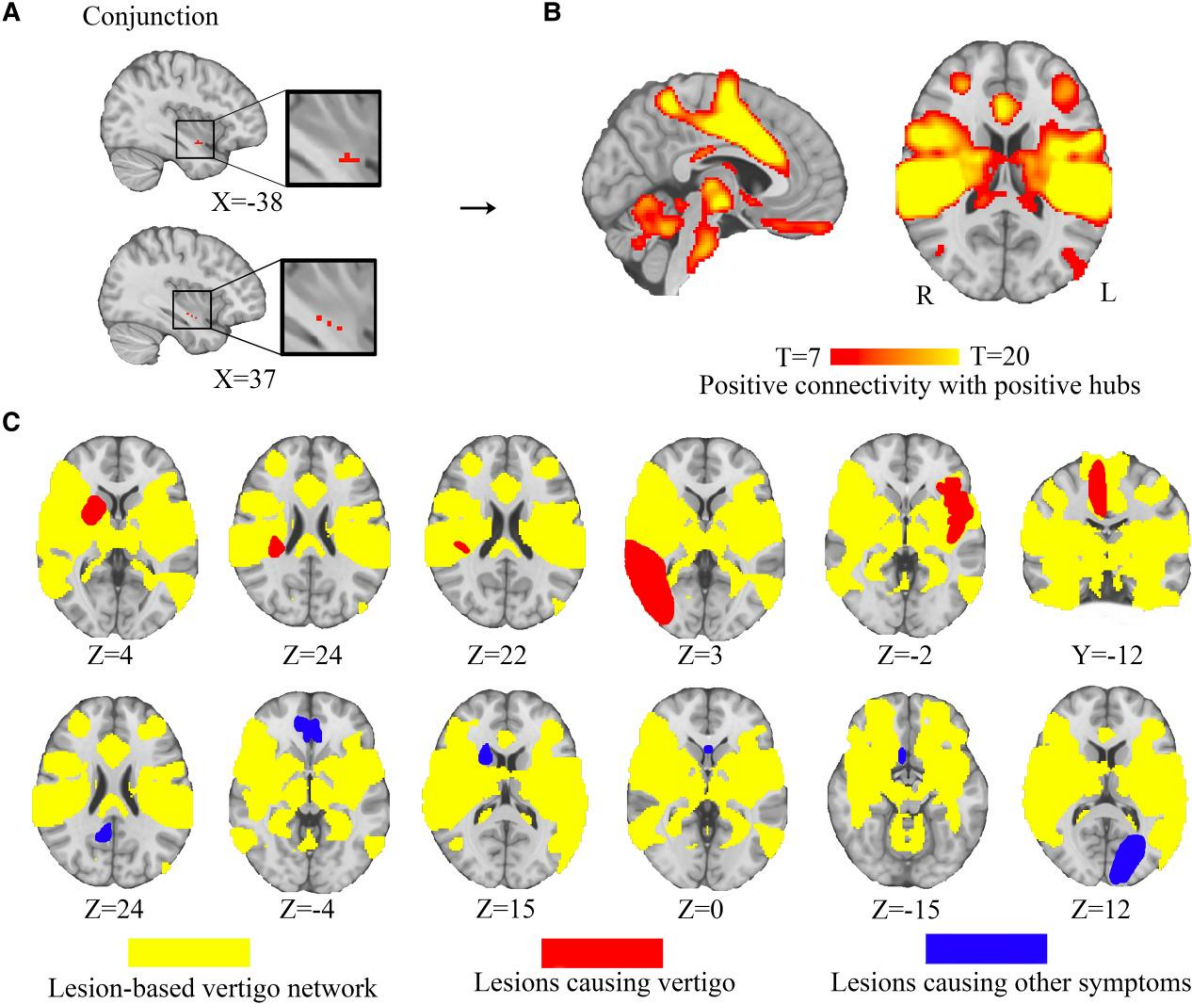
**A lesion-based vertigo network.** (**A**) The conjunction of the sensitivity and specificity test identifies the hub (bilateral posterior insular cortex) of the lesion-based vertigo network. (**B**) Functional connectivity with the hub defines a lesion-based vertigo network. (**C**) By definition, this lesion-based vertigo network encompasses heterogenous lesion locations associated with vertigo, but not control lesions not associated with vertigo.

We compared lesion network maps of vertigo with that of other symptoms (*n* = 68, Supplementary Fig. 3), and the results for specificity test were shown in Fig. 2D. The peak lesion network overlap in the bilateral ventral posterior insula was both sensitive (>96%) and specific (FWE-corrected *P* < 0.05) to lesions causing vertigo as revealed by conjunction analysis (Fig. 3A).

The network was defined by positive connectivity to lesion-based hub in bilateral ventral posterior insula. We shall refer to this network as lesion-based vertigo network (Fig. 3B). This network is bilaterally organized, predominately including bilateral PIC, OP2, somatosensory cortex, higher-level visual areas (visual area 5/middle temporal), cingulate sulcus visual area and thalamus. As expected, lesions associated with vertigo aligned well with the lesion-based vertigo network (96%), while lesions associated with other symptoms did not (Fig. 3C).

### Comparison of lesion-based vertigo network with cortical stimulation sites eliciting vertigo

**Figure 4:**
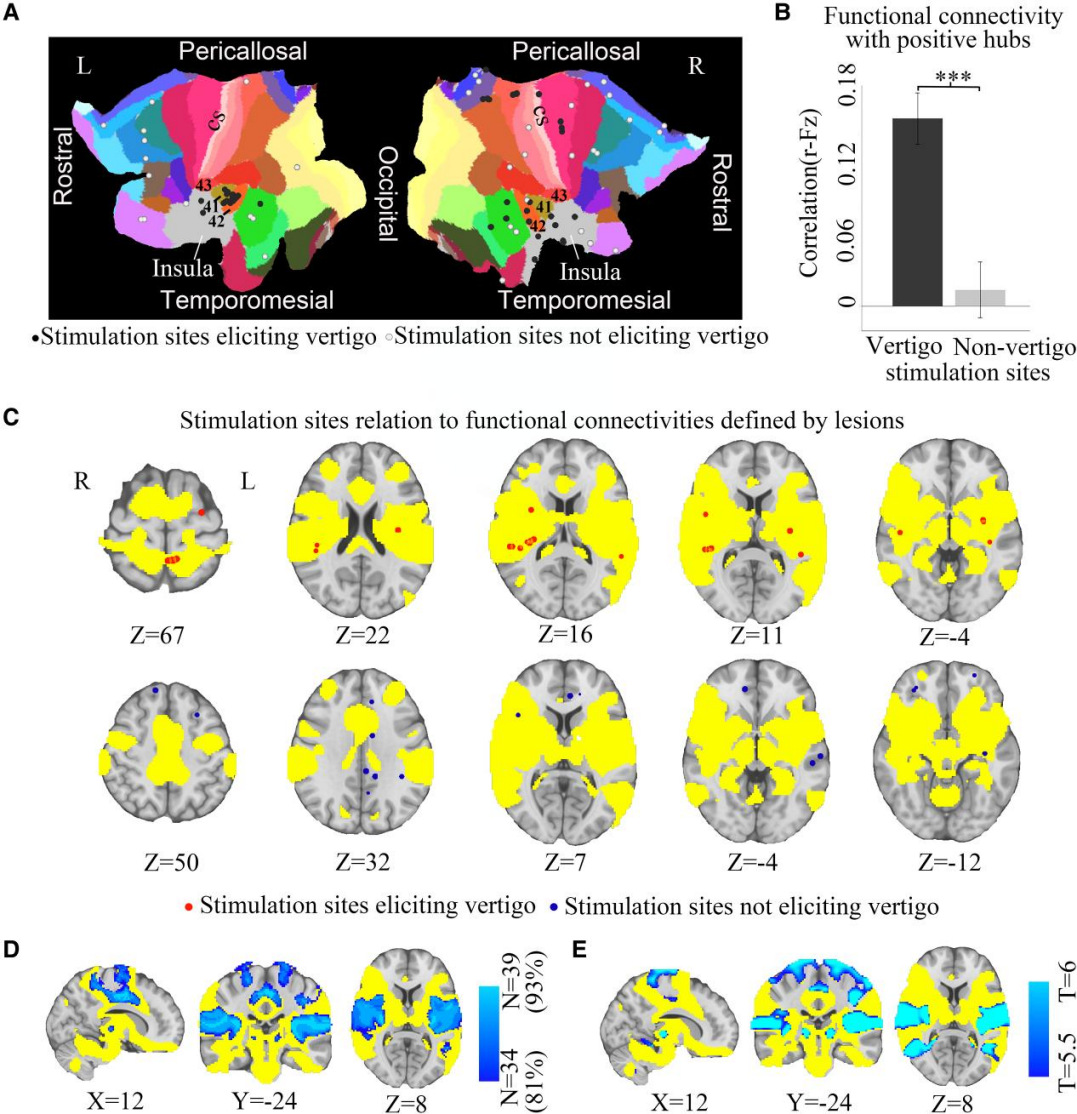
**Comparison of the lesion-based network with coordinate-based stimulation sites**. (**A**) Forty-two stimulation sites eliciting vertigo or not eliciting vertigo combined across 17 subjects overlaid on cortical flat map displaying Brodmann area for right and left hemispheres. The relationship of stimulation sites eliciting vertigo and insula is shown. (**B**) Connectivity of the stimulation sites to the hub of the lesion-based vertigo network is stronger for stimulation sites eliciting vertigo than stimulation sites not eliciting vertigo (two-sample *t*-test, ***P < 0.001). (**C**) Stimulation sites eliciting vertigo fall within the lesion-based vertigo network defined by brain lesions, while stimulation sites not eliciting vertigo do not fall within this network. (**D**) Stimulation-based vertigo network (sensitivity map, blue-green, shown thresholded at 34/39 stimulation sites) overlaid on the lesion-based vertigo network. (**E**) *T* test comparing connectivity of stimulation sites that did versus did not elicit vertigo (specificity map, blue-green, shown after voxel-wise FWE-corrected *P* < 0.05) overlaid on the lesion-based vertigo network.

We identified 42 distinct stimulation sites eliciting vertigo in 17 patients (11 males and 6 females, details in Supplementary Table 3). Similar to brain lesions causing vertigo, cortical stimulation sites eliciting vertigo were spatially distributed across multiple different brain regions, displayed on Brodmann areas shown in Fig. 4A. Only nine stimulation sites fell into PIC or OP2 (Supplementary Fig. 5). After reconstruction of stimulation sites and normalization into a standard MNI template, coordinate-based ROIs were generated centred on each stimulation sites with a 3-mm radius. The results of ROI-to-ROI functional connectivity analysis indicated that stimulation sites eliciting vertigo were more connected (two-sample *t*-test, *P* < 0.0001, Fig. 4B) to the lesion-based hub in bilateral ventral posterior insula compared to stimulation sites not eliciting vertigo. We found that 36/42 (86%) of stimulation sites eliciting vertigo fell within the lesion-based vertigo network in contrast to 16/39 (41%) of stimulation sites that did not elicit vertigo (Fig. 4C).

### DES coordinate-based sites network mapping

Identical to the lesion network mapping analyses using the same normative connectome, we computed a data-driven coordinate-based DES sites network. Both the sensitivity map (39/42, 93%, Fig. 4D) and the specificity map (Fig. 4E) derived from stimulation sites showed similar topography to our lesion-based vertigo network.

**Figure 5:**
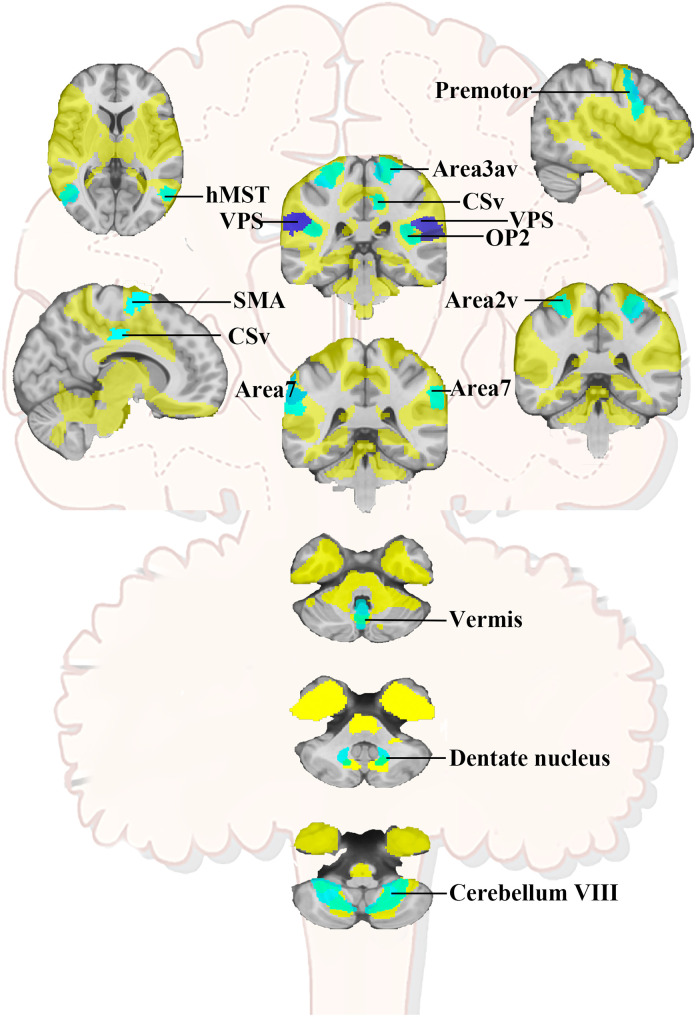
**Schematic illustration of the lesion-based vertigo network.** Regions include the bilateral Area 2v, Area 3av, Premotor, CSv, OP2, Area 7, VPS (or PIC), SMA, hMST, thalamus as well as vermis, dentate nucleus and cerebellum VIII. Area 2v, intraparietal sulcus with the postcentral sulcus; Area 3av, the fundus of the central sulcus; CSv, cingulate sulcus visual area; OP2, parietal opercula 2; Area 7, area supramarginalis; VPS, visual posterior Sylvian; PIC, posterior insular cortex; hMST, human medial superior temporal area; SMA, supplementary motor area).

In total, vertigo network was schematically depicted. It consisted of the core regions of 10 cortical regions redefined from previous vestibular stimulation studies known in primates for vestibular processing.^27^ Interestingly, the vertigo network also consisted of the key components of vestibulocerebellum including the vermis, bilateral dentate nucleus and cerebellum VIII within the posterior inferior cerebellar artery territory^28^ (Fig. 5).

## Discussion

In this study, we show that supratentorial brain lesions associated with vertigo occur in different brain locations but localize to a common brain network. We also show that stimulation sites associated with vertigo localize to this same network. These results lend insight into the neuroanatomical substrate of vertigo, particularly at the supratentorial network level.

The lesion-based vertigo network in our study is defined by connectivity to hub in the bilateral ventral posterior insula. This network is both sensitive and specific, encompassing 96% of lesion locations associated with vertigo and 86% of stimulation sites associated with vertigo while avoiding lesions and stimulation sites not associated with vertigo. Interestingly, our lesion network hub in the ventral posterior insula falls outside the traditional boundaries of the human ‘vestibular cortex’ in PIC and OP2.^6^ However, substantial evidence from human and non-human studies has shown that posterior insula plays an important role in integrating information including vestibular, visual and somatosensory information.^29^ The posterior insula has reciprocal connections with cortical widespread regions and also receives four brainstem pathways linking the vestibular nuclei.^30^ An interhemispheric connection of the posterior insula involving the perception of vertigo.^10,31^ Here, we show that functional connectivity to the posterior insular defines a brain network that includes brain lesions and stimulation sites associated with vertigo.^32^ We hereafter refer to this network as a human vertigo network.

The vertigo network not only encompasses primary vestibular cortex in PIC and OP2 but also includes regions outside this area, including somatosensory cortex, higher-level visual areas (visual area 5/middle temporal), cingulate sulcus visual area and thalamus. Somatosensory and visual system are thought to work together with vestibular cortex to regulate posture, gait and gaze.^8^ Specifically, given the known role of visual area 5/middle temporal region in sensing moving stimuli, the involvement of such region rather than the primary visual cortex in vertigo seems to be plausible.^33^ Of note, cingulate sulcus visual area involved in vertigo is consistent with a close interconnection of vestibular projections to the primary and secondary visual cortex observed in cats^34^ and monkeys,^35^ suggesting self-motion is perceived by coordinating vestibular,^36^ visual^37^ and locomotion-relevant proprioceptive information.^38^ Consistent with the known knowledge that thalamus serves as a unique relay station of vestibular input to cortex,^39^ our results support the view that not only the posterolateral thalamic nuclei but also the paramedian thalamus are the important ‘gatekeepers’ for vestibular information.^30^ Also, our results might account for the typically temporary characteristic of the vertigo from central dysfunction due to the potential compensation mechanisms of the multimodal network.

Interestingly, the vertigo network was derived only from supratentorial lesions, but it includes subtentorial regions of the cerebellar vermis, bilateral dentate nucleus and cerebellum VIII. Indeed, these regions are key components of the vestibulocerebellum with strong connections to the ipsilateral vestibular nuclei, which are all within the posterior inferior cerebellar artery territory.^40^ Clinically, lesions localized in these regions are more likely to be associated with vertigo.^41^ Cerebellar stroke may present with the acute vestibular syndrome, characterized by the acute vertigo and nystagmus lasting days to weeks.^42^ However, with the developments of neuroimaging and electrophysiological testing, markedly increasing numerous of isolated vertigo originating from cerebellar lesions was diagnosed in recent years.^43^ It should be noted that cortical lesions manifest with acute vertigo usually do not accompany with nystagmus and oscillopsia, which is in line with the observation from DES studies.^4^ Occasionally, transient and slight nystagmus and oscillopsia can be observed in the initial stage, suggesting a highly robust and redundant vestibular cortical network re-organization.

In addition, we also demonstrate that brain lesions associated with vertigo and brain stimulation sites eliciting vertigo map to a common brain network. DES is a well-established technique to directly evaluate brain function. Compared with the previous studies of DES eliciting vertigo, we performed with a more rigorous inclusion criterion: only patients who reported they felt spinning of either the body or the surroundings were considered as vertigo, and only the symptoms elicited at the lowest intensity for a given DES site, which minimized the hybrid effects of the associated symptoms and maximized the detection of sites can elicit vertigo. Cortical stimulation sites eliciting vertigo were also spatially distributed across multiple different brain regions but localized in a common brain functional network. The convergence of the stimulation sites causing vertigo with lesion-based vertigo network validates the reliability of this method, which is in accordance with the findings of prior work that also used the similar protocol.^44^

Limitations of the present study are as follows. Firstly, it should be highlighted that we derived our vertigo network by retrospective literature review of rare, anecdotal case studies of lesions associated with vertigo. We are thus limited by the heterogeneous symptomatic characterization that was performed in these case studies, which could lead to confounding. For example, while lesions may link to vertigo, they may also associate with migraine, which could lead to the confusion of symptoms documentation. Of note, the clinical phenotyping was limited to the information available in the published case reports mainly from the subjective statements of patients themselves rather than objective measurements, restricting the ability to strictly exclude confounding factors. Future prospective studies with standardized assessment of vertigo symptoms and objectives measures of vestibular perception are needed to validate our findings. Secondly, we used a large normative functional connectivity data set to approximate the lesion connectivity in an individual patient, which ignores individual differences. However, these individual differences are small relative to the overall common connectivity pattern across subjects and prior works have suggested the little impact of age-matched or disease-matched connectome on the lesion network mapping results.^17^

Overall, we identify a human vertigo network based on brain lesions and brain stimulation and provide novel insights into the neuroanatomical network organization of vertigo.

## Supplementary Material

fcad071_Supplementary_Data

## Data Availability

The original data that support the findings of this study are available in Supplementary materials. All codes used to analyse the data are openly available within Lead-DBS/-Connectome software (https://www.lead-dbs.org/).

## Abbreviations

DES =: direct electrical stimulation
FWE =: family-wise error
MNI =: Montreal Neurological Institute
OP2 =: parietal opercula 2
PIC =: posterior insular cortex
PIVC =: parieto-insular vestibular cortex
ROI =: region of interest
SEEG =: stereo-electroencephalography
VPS =: visual posterior Sylvian
