# Supplementary material for: A vertigo network derived from human brain lesions and brain stimulation
Source: Brain Commun. 2023 Mar 17;5(2):fcad071. doi: 10.1093/braincomms/fcad071 (PMC10087025; doi:10.1093/braincomms/fcad071)
Supplement: fcad071_Supplementary_Data [file fcad071_Supplementary_Data.pdf]

Supplementary Figure 1

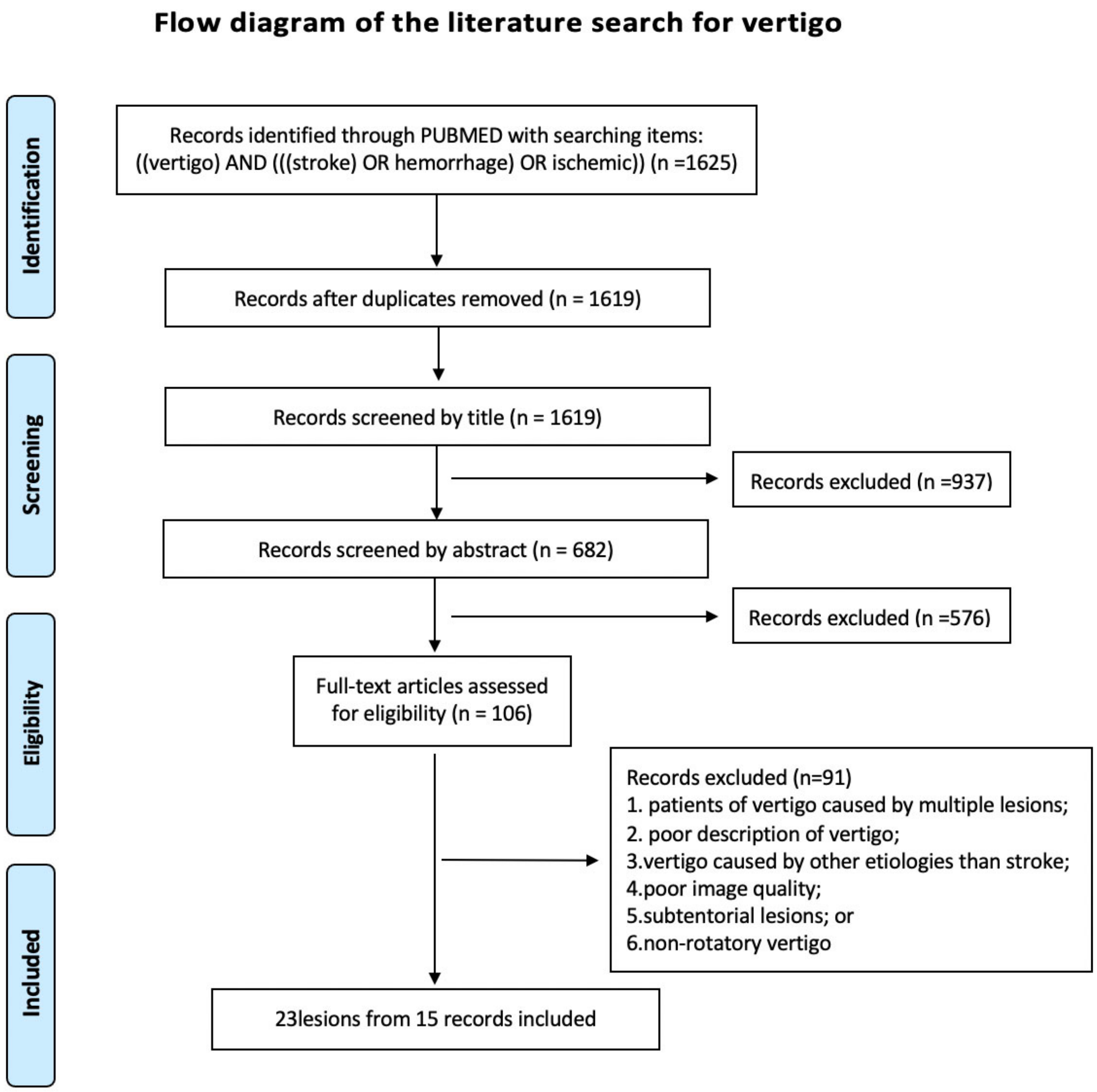

Supplementary Figure 1: Work flow for identifying cases of vertigo lesions from the literature. In total, 1619 articles matched initial search criteria. 937 articles were excluded after reviewing the title. Of this, 682 abstracts were reviewed and 576 were rejected as they did not meet the inclusion criteria. This left 106 articles for full-text review with exclusion criteria: (1) vertigo caused by multiple lesions or not limited (focal brain) lesions; (2) Lesions could not be reliably localized because of poor image quality; (3) subtentorial lesions; (4) vertigo associated with seizures or epileptiform discharge on EEG or (5) non-rotatory vertigo. Our final set totaled 23 lesions in 15 articles, which is intended to be a representative but not exhaustive set of lesion locations causing vertigo.

Supplementary Figure 2

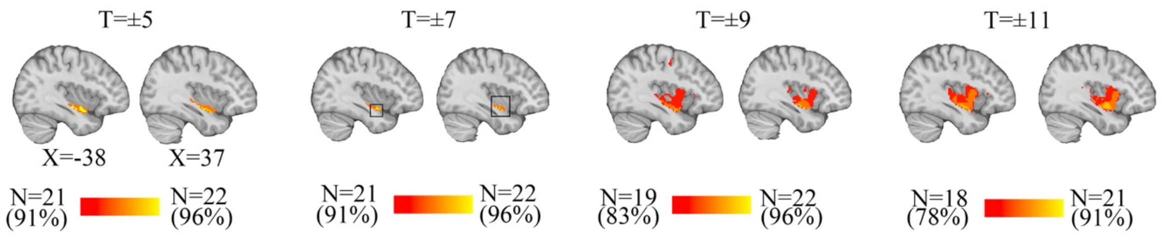

Supplementary Figure2: Lesion network mapping of findings are independent of threshold.

Regions of positive hubs including the bilateral insular cortex, which are similar across different network thresholds.

Supplementary Figure 3

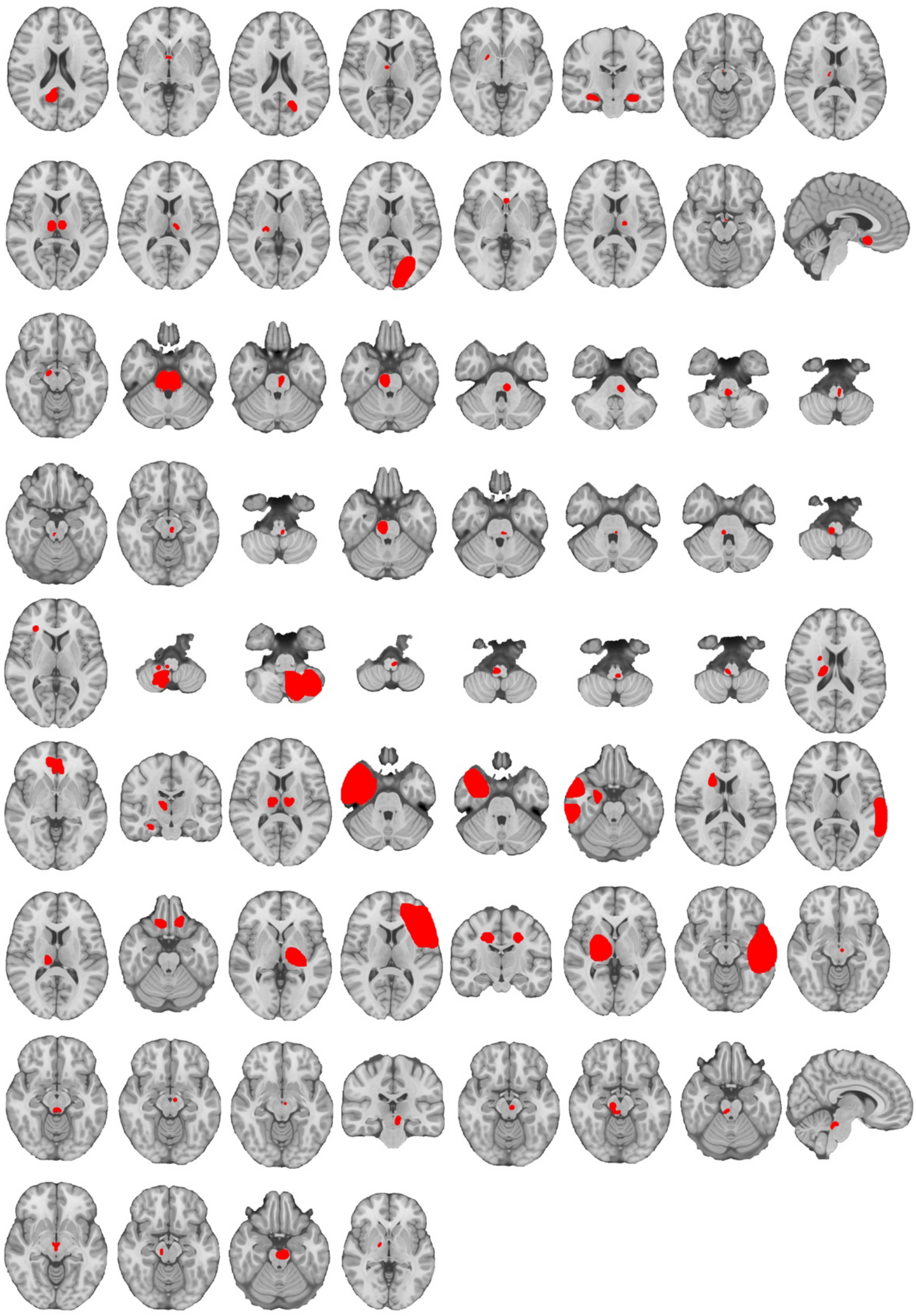

Supplementary Figure3: Lesion locations associated with other symptoms of the control group.

To test for specificity, we compared our lesion locations associated with vertigo to 68 lesions causing other symptoms including 20 lesions causing facial palsy, 17 lesions causing amnesia,

19 lesions causing mania and 12 lesions causing Parkinson's disease. The locations of all the control lesions are spatially heterogeneous over supratentorial and subtentorial structures.

Supplementary Figure 4

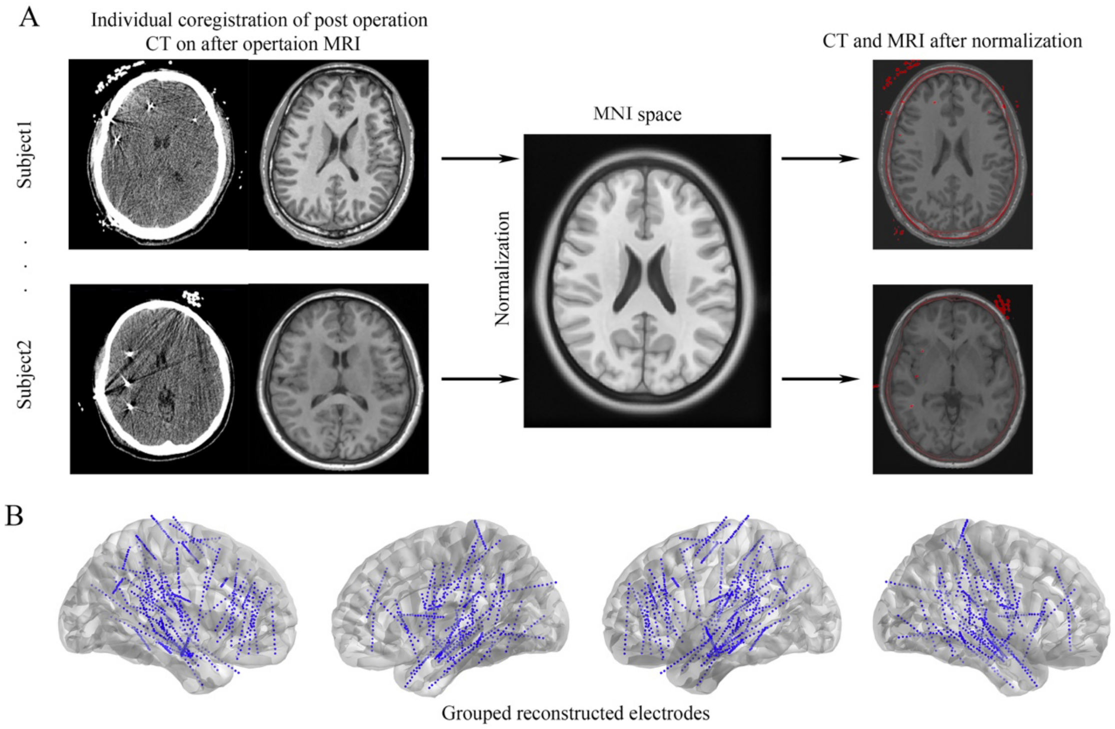

Supplementary Figure4: Procedure of localizing the SEEG electrodes.

(A) Displayed the co-register and normalization procedure between after operation MRI and post operation CT of patients. (B) All implanted electrodes of 17 patients overlaid on a standardized brain in MNI space.

Supplementary Figure 5

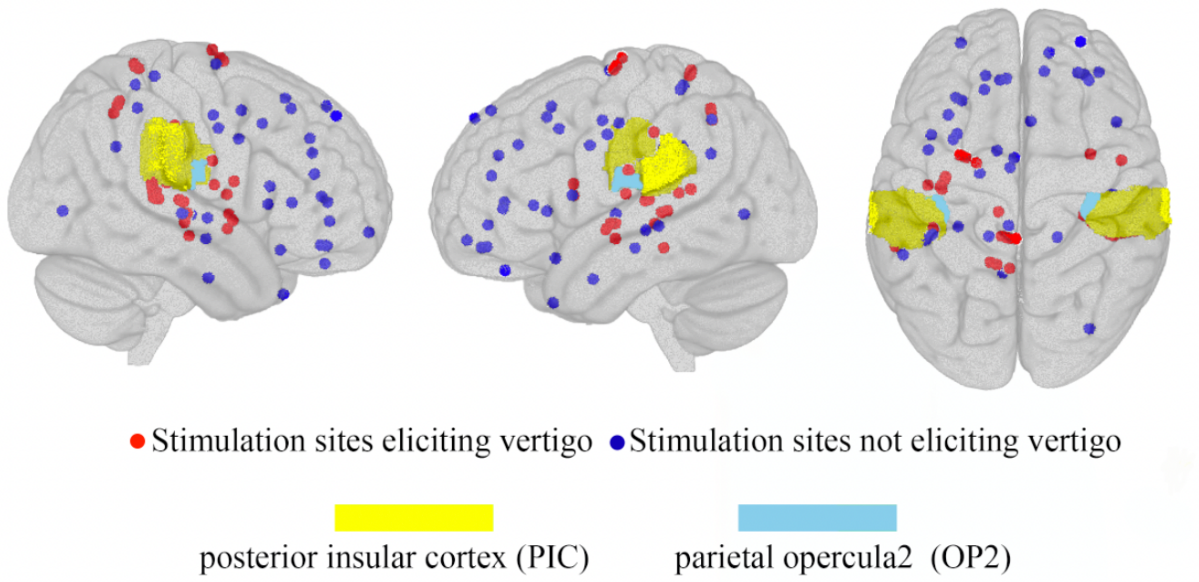

Supplementary Figure 5: Relationship of DES sites with the positive hub of human vertigo network in MNI brain.

42 stimulation sites eliciting vertigo (red) or not eliciting vertigo (blue) combined across 17 subjects, displayed on the standard MNI brain. The relationship of stimulation sites eliciting vertigo and PIC (yellow) and OP2 (azure) is shown.

Supplementary Table 1: References for the 23 case studies meeting inclusion criteria and exclusive criteria for our analysis, taken from 15 unique journal articles. All 23 lesion cases were classified as vertigo causing by stroke.

Patient Number	Gender	Symptom	Causes of Lesions	Literature	Original Imaging
NO.1	Female	rotational vertigo	stroke	von Brevern, M., et al. (2014). "Acute vertigo due to hemispheric stroke." Journal of the Neurological Sciences 339(1-2): 153-156.	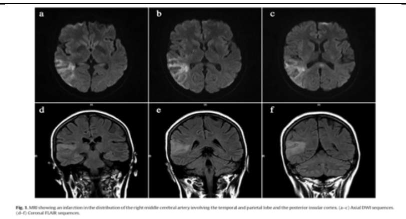
NO.2	Female	rotational vertigo	stroke	Ahn, B.-Y., et al. (2010). "Pseudovestibular neuritis associated with isolated insular stroke." Journal of Neurology 257(9): 1570-1572.	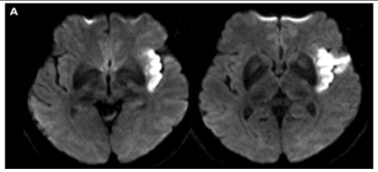
NO.3	Female	vertigo	hemorrhagic stroke	Moore, P., et al. (2018). "Hemorrhagic stroke after Epley maneuver: a case report." Journal of Otolaryngology - Head & Neck Surgery 47(1).	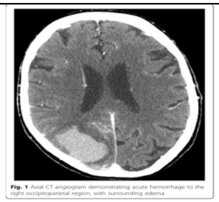
NO.4	Female	positional vertigo	stroke	Naganuma, M., et al. (2006). "Rotational vertigo associated with parietal cortical infarction." Journal of the Neurological Sciences 246(1-2): 159-161	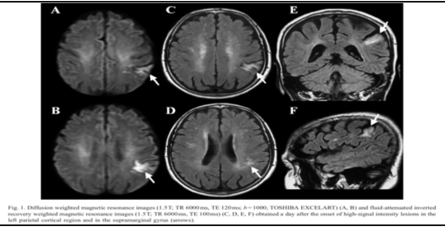

NO.5	Male	rotational vertigo	stroke	Nakajima, M., et al. (2012). "Rotational Vertigo Associated with Putaminal Infarction." Journal of Stroke and Cerebrovascular Diseases 21(8): 912.e919-912.e910	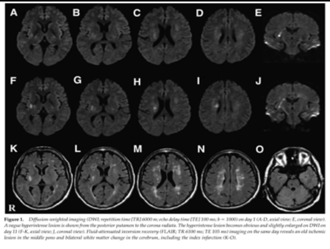
NO.6	Male	vertigo	stroke	Park, K. M., Shin, K. J., Ha, S. Y., Park, J. & Kim, S. E. Isolated rotational vertigo due to internal capsular infarction. *J. Neuro-Ophthalmology* **34**, 61–63 (2014).	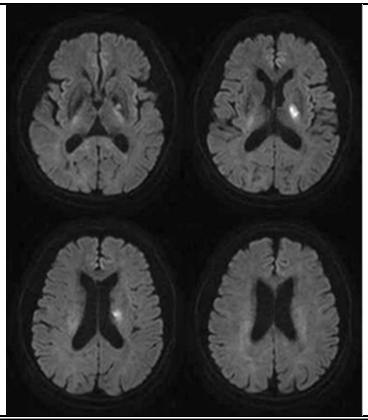
NO.7	Female	vertigo	stroke	Kim, H. A., Lee, S. R. & Lee, H. Acute peripheral vestibular syndrome of a vascular cause. J. Neurol. Sci. 254, 99–101 (2007).	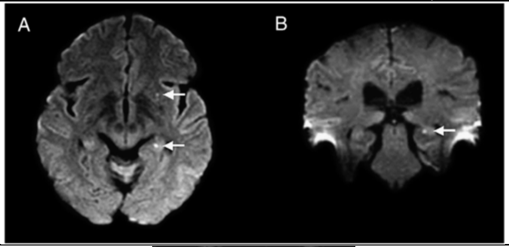
NO.8	Female	isolated vertigo	stroke	Kim, H. A. and H. Lee (2012). "Recent advances in central acute vestibular syndrome of a vascular cause." J Neurol Sci 321(1-2): 17-22.	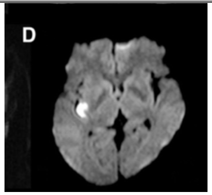

NO.9	Female	rotatory vertigo	hemorrhage	J Boiten, J. W., H Kingma (2003). "Acute rotatory vertigo caused by a small haemorrhage of the vestibular cortex." J Neurol Neurosurg Psychiatry: 388-394.	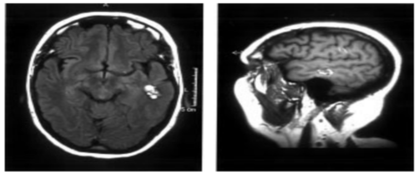<br>**Figure 1** Transverse T2 weighted, fluid attenuated inversion recovery (FLAIR) image (left) and sagittal T1 weighted spin echo image (right). Both show a small popcorn shaped area of increased signal intensity demarcated by a rim of decreased signal intensity (haemosiderin), located in the left medial temporal gyrus. The combination of recent blood products and older haemorrhagic residues is consistent with the diagnosis of a cavernous haemangioma.
NO.10	Female	vertigo	stroke	Park, K. M. et al. Isolated foot drop in acute infarction of the supplementary motor area. Clin. Neurol. Neurosurg. 115, 2240–2242 (2013).	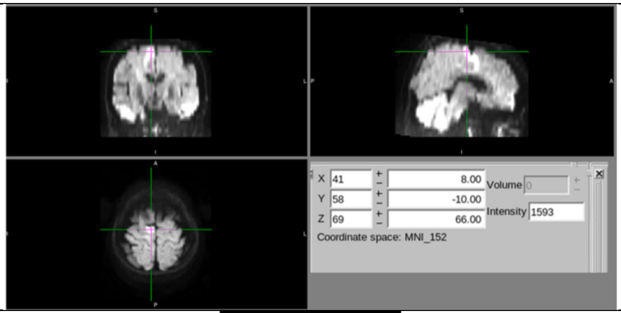
NO.11	Male	rotational vertigo	stroke	S. Debette a, E. M. b., H. Hénon a, D. Leys a (2003). "Transient Rotational Vertigo as the Initial Symptom of a Middle Cerebral Artery Territory Infarct Involving the Insula." Cerebrovasc Dis: 97-98.	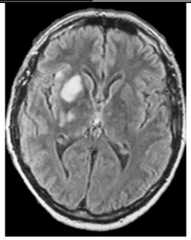
NO.12	Female	rotational vertigo	stroke	Th. Brandt, M. K. B., MD; T. Yousry, MD; M. Dieterich, MD; and S. Schulze, MD (1995). "Rotational vertigo in embolic stroke of the vestibular and auditory cortice." NEUROLOGY: 42-44.	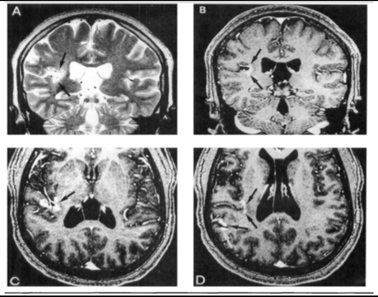

NO.13	Female	vertigo	stroke	Farheen Niazi1 and Sameen Bin Naeem2. Artery of Percheron Infarct: A Rarity Not to be Missed. 577–578 (2017).	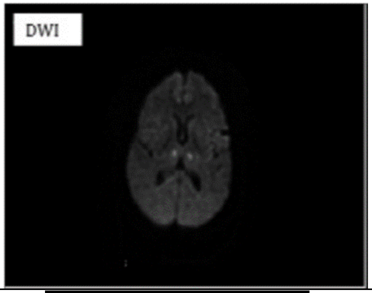
NO.14	Male	vertigo	stroke	Eguchi, S., Hirose, G. & Miaki, M. Vestibular symptoms in acute hemispheric strokes. J. Neurol. (2019). doi:10.1007/s00415-019-09342-9	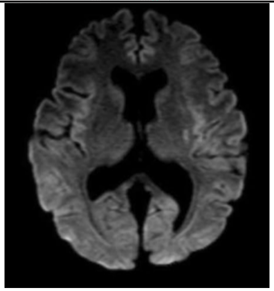
NO.15	Male	vertigo	stroke	Eguchi, S., Hirose, G. & Miaki, M. Vestibular symptoms in acute hemispheric strokes. J. Neurol. (2019). doi:10.1007/s00415-019-09342-9	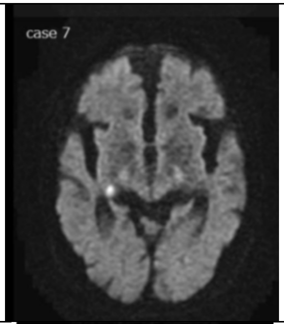
NO.16	Male	vertigo	stroke	Eguchi, S., Hirose, G. & Miaki, M. Vestibular symptoms in acute hemispheric strokes. J. Neurol. (2019). doi:10.1007/s00415-019-09342-9	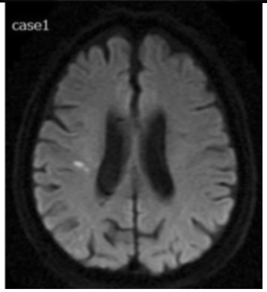

NO.17	Male	vertigo	stroke	Eguchi, S., Hirose, G. & Miaki, M. Vestibular symptoms in acute hemispheric strokes. J. Neurol. (2019). doi:10.1007/s00415-019-09342-9	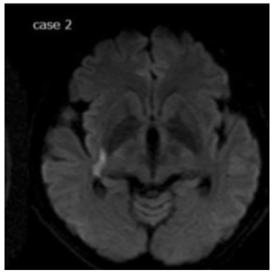
NO.18	Female	vertigo	stroke	Eguchi, S., Hirose, G. & Miaki, M. Vestibular symptoms in acute hemispheric strokes. J. Neurol. (2019). doi:10.1007/s00415-019-09342-9	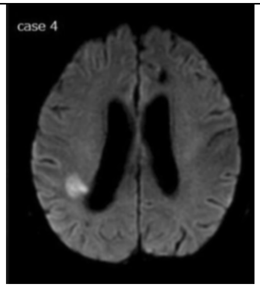
NO.19	Male	vertigo	stroke	Eguchi, S., Hirose, G. & Miaki, M. Vestibular symptoms in acute hemispheric strokes. J. Neurol. (2019). doi:10.1007/s00415-019-09342-9	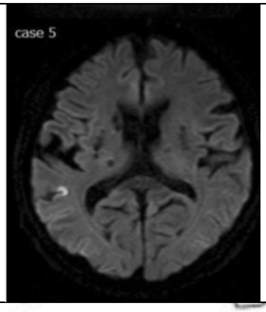
NO.20	Female	vertigo	stroke	E., K., T., O. & Y., A. Strokes in the subinsular territory: Clinical, topographical, and etiological patterns. *Neurology* **63**, 2429–2432 (2004).	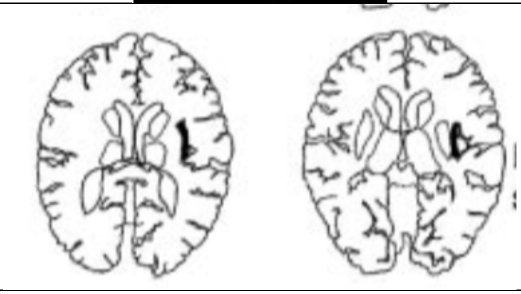

NO.21	Male	vertigo	stroke	E., K., T., O. & Y., A. Strokes in the subinsular territory: Clinical, topographical, and etiological patterns. *Neurology* **63**, 2429–2432 (2004).	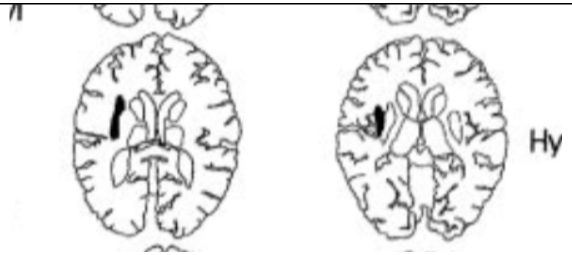
NO.22	Female	vertigo	stroke	E., K., T., O. & Y., A. Strokes in the subinsular territory: Clinical, topographical, and etiological patterns. *Neurology* **63**, 2429–2432 (2004).	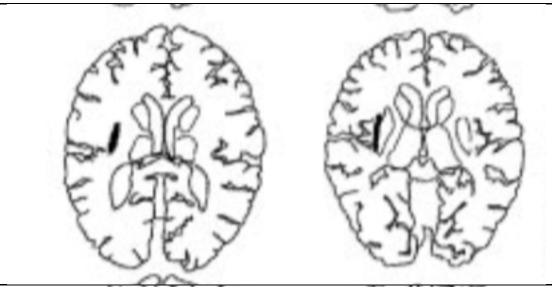
NO.23	Male	vertigo	stroke	E., K., T., O. & Y., A. Strokes in the subinsular territory: Clinical, topographical, and etiological patterns. *Neurology* **63**, 2429–2432 (2004).	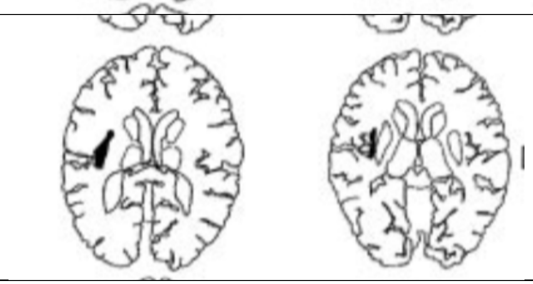

Supplementary Table 2: The original description of vertigo from the literatures

Patient number	Original description
1	A 51-year-old woman with a history of migraine without aura awoke at night with constant rotational vertigo, oscillopsia, slight nausea and severe imbalance[1].
2	Her vertigo occurred regardless of position change, which was not accompanied by nausea or vomiting. Neurological examinations on that day showed spontaneous right beating horizontal nystagmus[2].
3	The symptoms of vertigo, severe nausea, and blurred vision persisted after 30 min and the patient was transferred to the emergency department for emergent assessment, including CT angiogram of the brain[3].
4	A 65-year-old healthy woman suddenly experienced rotational vertigo soon after she rotated her neck slowly to the right side while sitting in her chair around midnight.<br>Her vertigo could be provoked by rotating her head in any direction. The patient's eye movements were full but she displayed spontaneous nystagmus to the right side when her eyes were in their primary position. She also displayed spontaneous nystagmus to the right side in all eye positions including vertical and horizontal gaze changing, whether or not her head was moving[4].
5	The patient suddenly complained of rotational vertigo and vomited. He displayed spontaneous nystagmus to the left side in all eye positions[5].
6	A right-handed 64-year-old man reported acute onset rotational vertigo with unsteady gait. The vertigo was continuous and not affected by changes in position. He complained of nausea and vomiting but denied tinnitus, hearing loss, altered speech, sensory disturbance, or muscle weakness. Examination revealed spontaneous horizontal right-beating horizontal nystagmus with a torsional component in primary and eccentric positions of gaze, even during visual fixation[6].
7	A 67-year-old woman experienced abrupt onset (evolved over several minutes) of severe vertigo, nausea, and vomiting, which lasted for 3 days. On neurological examination performed three days after onset of vertigo, she had a spontaneous right-beating horizontal nystagmus with a torsional component, in the primary position and with gaze to the right or left. She showed corrective saccadic movements during head thrust testing toward the left side[7].
8	isolated vertigo[8]
9	A 53-year-old woman suddenly experienced leftwards directed rotatory vertigo in the yaw plane and nausea without vomiting. Examination of the cranial nerves showed no abnormalities; specifically, there was no nystagmus or hearing loss and the eye movements were normal[9].
10	She noted transient vertigo of a whirling type for approximately 30 s initially and then sudden development of weakness in the left foot. She was alert and oriented upon neurologic examination. No cranial nerve function deficits were observed[10].
11	He suddenly experienced a severe rotational vertigo with nausea[11].

12	Suddenly experienced rotational vertigo with a subsequent fall to the left, nausea, and vomiting. She also noticed slight numbness and dysesthesia on the left side of her face and body[12].
13	Symptoms started suddenly when she woke up in the morning. She was conscious and oriented in time, place and person. Examination of the ocular movements showed complete vertical gaze palsy. The patient was afebrile and no meningeal signs were present[13].
14	vertigo, nausea no nystagmus[14]
15	vertigo, nausea head-motion dizziness no nystagmus[14]
16	vertigo, vomiting<br>gaze nystagmus to right > left[14]
17	vertigo, vomiting unsteadiness on walking no nystagmus[14]
18	vertigo, nausea no nystagmus[14]
19	vertigo, nausea unsteadiness on walking no nystagmus[14]
20	vertigo, anosognosia, dysphagia, dysarthria, hemiplegia[15]
21	Numbness, vertigo, apathy[15]
22	Numbness, vertigo[15]
23	Numbness, vertigo, TMA (transient-motor aphasia), dysphagia[15]

Supplementary Table 3: Direct electrical stimulation sites (N=42) eliciting vertigo and their anatomical locations in patient (N=17) underwent SEEG monitoring.

Patient number	Gender	Age	Location of stimulation sites eliciting vertigo (MNI coordinates)		
			X	Y	Z
Pat1	male	26	-41	-9	-9
Pat2	male	35	-46	-30	3
			-48.5	-32.5	4
Pat3	female	24	-45.5	-38	12
Pat4	male	42	41	-31	7
			42	-32	10.5
			42.5	-33.5	13.5
			43.5	-34.5	17
			44.5	-36	20
Pat5	male	32	-9.5	-30	39.5
Pat6	male	29	33	3	15
Pat7	female	18	43	-33	7.5
			45.5	-33	9.5
			48.5	-33	12
			51	-33	14.5
			54	-33	16.5
Pat8	male	25	-39.5	-27.5	-2
			-62	-29.5	-13
			-56	-42.5	14
Pat9	male	6	-35	-14.5	11.5

			-36	-15.5	22.5
Pat10	female	26	-2	-48	68
			-5.5	-47.5	66.5
			-8.5	-46.5	65.5
			-21	-14	74
			-26	-11	70.5
			-28.5	-9.5	68.5
			-33	-5.5	-5
			-33.5	-7.5	-2
Pat11	female	21	-4	-57.5	45
			-10	-56.5	48.5
			-13	-56	50.5
Pat12	male	25	49	-18	-3.5
Pat13	male	35	34.5	-29.5	16.5
Pat14	male	28	47.5	1.5	9.5
Pat15	female	19	-39	-36.5	27.5
Pat16	female	18	47	-28	19
Pat17	male	12	30.5	-26	16
			33.5	-27.5	17
			37	-28.5	18
			40	-29.5	19
			43.5	-30.5	20

Reference

1. von Brevern M, Süßmilch S, Zeise D. Acute vertigo due to hemispheric stroke: a case report and comprehensive review of the literature. *J Neurol Sci*. Apr 15 2014;339(1-2):153-6.
2. Ahn BY, Bae JW, Kim DH, Choi KD, Kim HJ, Kim EJ. Pseudovestibular neuritis associated with isolated insular stroke. *J Neurol*. Sep 2010;257(9):1570-2.
3. Moore P, Le T, Blakley B, Beiko J, Meen E. Hemorrhagic stroke after Epley maneuver: a case report. *J Otolaryngol Head Neck Surg*. Apr 10 2018;47(1):25.
4. Naganuma M, Inatomi Y, Yonehara T, et al. Rotational vertigo associated with parietal cortical infarction. *J Neurol Sci*. Jul 15 2006;246(1-2):159-61.
5. Nakajima M, Inatomi Y, Yonehara T, Hirano T, Uchino M. Rotational vertigo associated with putaminal infarction. *J Stroke Cerebrovasc Dis*. Nov 2012;21(8):912.e9-10.
6. Park KM, Shin KJ, Ha SY, Park J, Kim SE. Isolated rotational vertigo due to internal capsular infarction. *J Neuroophthalmol*. Mar 2014;34(1):61-3.
7. Kim HA, Lee SR, Lee H. Acute peripheral vestibular syndrome of a vascular cause. *J Neurol Sci*. Mar 15 2007;254(1-2):99-101.
8. Kim HA, Lee H. Recent advances in central acute vestibular syndrome of a vascular cause. *J Neurol Sci*. Oct 15 2012;321(1-2):17-22.
9. Boiten J, Wilmink J, Kingma H. Acute rotatory vertigo caused by a small haemorrhage of the vestibular cortex. *J Neurol Neurosurg Psychiatry*. Mar 2003;74(3):388.
10. Park KM, Kim SE, Shin KJ, et al. Isolated foot drop in acute infarction of the supplementary motor area. *Clin Neurol Neurosurg*. Oct 2013;115(10):2240-2.
11. Debette S, Michelin E, Hénon H, Leys D. Transient rotational vertigo as the initial symptom of a middle cerebral artery territory infarct involving the insula. *Cerebrovasc Dis*. 2003;16(1):97-8.
12. Brandt T, Bötzel K, Yousry T, Dieterich M, Schulze S. Rotational vertigo in embolic stroke of the vestibular and auditory cortices. *Neurology*. Jan 1995;45(1):42-4.
13. Niazi F, Naeem SB. Artery of Percheron Infarct: ARarity Not to be Missed. *J Coll Physicians Surg Pak*. Sep 2017;27(9):577-578.
14. Eguchi S, Hirose G, Miaki M. Vestibular symptoms in acute hemispheric strokes. *J Neurol*. Aug 2019;266(8):1852-1858.
15. Kumral E, Ozdemirkiran T, Alper Y. Strokes in the subinsular territory: clinical, topographical, and etiological patterns. *Neurology*. Dec 28 2004;63(12):2429-32.
